# Parkinson's Disease and Forced Exercise: A Preliminary Study

**DOI:** 10.1155/2013/375267

**Published:** 2013-06-19

**Authors:** Abu Qutubuddin, Timothy Reis, Raed Alramadhani, David X. Cifu, Alan Towne, William Carne

**Affiliations:** ^1^Richmond Veterans Affairs Medical Center, Southeast Parkinson's Disease Research, Education and Clinical Center, 1220 Broad Rock Boulevard, Richmond, VA 23249, USA; ^2^Department of PM&R, Virginia Commonwealth University, 1223 East Marshall Street, Richmond, VA 23298, USA; ^3^Department of Neurology, Virginia Commonwealth University, P.O. Box 980599, Richmond, VA 23298-0599, USA

## Abstract

*Objective*. The concept of forced exercise has drawn attention for the treatment of Parkinson's disease symptoms with anecdotal reports of success. This study sought to ascertain any significant effect of forced exercise using a motorized stationary bicycle when compared to controls on Parkinson's disease symptoms in a blinded, randomized, and controlled setting. *Setting*. Parkinson's disease outpatient clinic, Veterans Administration Medical Center. *Method*. We assessed 23 patients (13 experimental and 10 controls) on a number of standard Parkinson's measures at baseline, after participation in eight weeks of twice weekly forced exercise or eight weeks of conventional clinic care, and then after a three-month period had elapsed. Dependent measures were UPDRS-III, Berg Balance Scale, finger taping test, and the PDQ-39. *Results*. Results did not demonstrate any main effect differences between the exercise and control groups on any measure at any point in time. A within subjects effect was demonstrated for the forced exercise group on overall UPDRS-III scores at the three-month end point. No other within group effects were noted. Results suggest that early enthusiasm for forced exercise may need tempering. Limitations of the study are discussed as well as numerous logistical challenges to this type of study.

## 1. Introduction

Exercise therapy is well known to be beneficial for the elderly in general [1–4], and more recently the benefit of exercise for patients with Parkinson's disease (PD) has been investigated [5–8]. The exact pathophysiological mechanism of exercise and types of specific exercises beneficial to persons with PD is still open to question [9]. Although there are limited exercise studies involving persons with Parkinson's [10], some [11] postulate that the benefit of exercise is associated with synaptic plasticity, cascading effect of growth factors, and by reducing ancillary conditions (e.g., hypertension) that may adversely impact the brain.

One limiting factor in studying the effect of exercise has been the ability of the patient with PD to maintain sufficient exercise intensity to produce appreciable benefit to the patient. Exercise intensity in this population can be limited by a number of factors to include bradykinesia and imbalance as well as general deconditioning. Recent studies [12, 13] have tried to overcome these limiting factors by the use of tandem bicycles (where the patient is placed in the rear seat and a fit trainer in the front seat maintains a strict cadence of at least 80 revolutions-per-minute (rpm)). Thus the person with PD is “forced” to maintain a higher than usually achievable level of exercise. Even more recently, motorized single seat stationary bicycles have been used for individuals with PD to maintain a forced-exercise (FE) level without the need of a trainer [14].

Researchers [12] have examined the impact of FE on the Unified Parkinson's Disease Rating Scale (UPDRS) Part III (motor component) scores and on bimanual dexterity grasping tasks. They found that both the voluntary exercise and forced exercise groups improved aerobically but only the FE group experienced an improved UPDRS score and better bilateral manual dexterity.

Persons with PD and Parkinson's researchers alike have been intrigued by the relatively recent news of a possible relationship between PD motor symptoms and bicycling. A Danish researcher found that bicycling is a preserved motor activity for persons with PD [13] while others have noted the beneficial impact of relatively high intensity cycling on tremors [12]. However due to the relative freshness of these findings, limited formal studies have been conducted. 

Consequently, our group conducted a small sample study to examine the impact of cycling on various Parkinson's symptoms with the intent to expand the study to a much larger sample in the next phase. In addition to examining the impact of cycling FE on PD symptoms in a more systemized manner, we also investigated the difficulties that could be anticipated if conducting a rigorous, large-scale study of cycling and PD. 

## 2. Methods

Following local Institutional Review Board approval of the study, subjects were recruited thorough hospital and clinic advertisements at the Southeast Parkinson's Disease Research, Education and Clinical Center (PADRECC) of the Richmond, Virginia Veterans Affair Medical Center. All subjects had a three-year confirmed PD diagnosis with good response to standard Parkinson's medications and a UPDRS Part III score of >30. Exclusion criteria were nonambulating patients, demented patients, patients already enrolled in an on-going PD drug research study, patients with uncontrolled diabetes or hypertension, patients with chronic obstructive pulmonary disease, and a history of coronary artery disease or congestive heart failure.

 After fully informed consent was obtained, 23 subjects were randomly assigned to either an exercise group (*n* = 13) or a control group (*n* = 10). All subjects were then evaluated at baseline using the Berg Balance Scale (BBS) [15], Unified Parkinson's Disease rating Scale, Part III (UPDRS-III) [16], finger taping test [17], and Parkinson's Disability Questionnaire (PDQ-39) [18, 19].

Control and exercise subjects received usual PADRECC clinic care involving medical visits (nursing, neurology, physiatry, neuropsychology, and family support services) as indicated and appropriate medication changes as necessary. Those in the control group received no specialized physical therapy or exercise conditioning. Exercisers underwent an eight-week, twice weekly, cycling program with sessions consisting of warm-up, sustained 30 minute exercise, and cool down, for a total of 16 FE sessions. Exercisers and controls were reevaluated at the eight-week immediate postexercise interval and then again after four months of exercise completion. 

The Theracycle (RSS Industries, Inc., Franklin, MA, USA) is a stationary bicycle with motorized pedals that can be programmed to move at a selected speed without effort by the rider. There are also moving handlebars designed to provide an upper body exercise component. The handlebars were disabled and not used in this study as it was felt that the intensity could be too intimidating for some subjects. FE intensity was defined as 61–80% of the individual's aerobic maximum target heart rate. Target heart rates were calculated using the Karnoven formula (220-minus patient age = maximal heart rate) [14]. Each FE participant participated in two supervised sessions per week (Monday/Thursday or Tuesday/Friday) of FE for eight consecutive weeks. Sessions consisted of a warm-up period, followed by a 30-minute FE period in which the targeted heart rate was maintained, followed by a cool down period. While in the FE phase participants' heart rates were monitored by a trained research assistant using an Omron HR-100C Heart Rate Monitor (Omron Healthcare, Inc., Lake Forest, IL, USA).

On the day of and prior to exercise initiation, all subjects were rated by a blinded neurologist using the UPDRS Part III. Blinded evaluators administered the BBS as well. Evaluators, all of whom had a bachelor's or higher degree, were specially trained by the principal investigator in administering these tests. In addition a blinded neuropsychologist administered the finger taping test for both dominant and nondominant hand. Finally all participants completed the PDQ-39. Participants were evaluated using the same measures at the end of the eight-week exercise period by the same blinded raters. A third and final rating was administered four months after the completion of exercise. Each evaluation took less than 45 minutes.

All evaluations were conducted while the subjects were in the “on” state (within 3 hours) of medication. The “on” state refers to the pulsatile nature of PD medications and wearing off of effectiveness.

### 2.1. Measures

The BBS is an objective measure of balance abilities. It has been used to identify and evaluate balance impairment. The scale consists of 14 tasks common in everyday life. The items test a subject's ability to maintain positions or movements of increasing difficulty by diminishing the base of support from sitting and standing to single-leg stance. One's ability to change positions is also assessed.

The UPDRS is currently the most widely accepted scale for measuring the different components of PD. It is used in clinical research and drug trials to follow the longitudinal course of PD. Its major strength is that it provides a detailed and accurate assessment of PD in different respects. It is divided into 6 sections. For this study, we only used the scale's motor component (Part III). This is a detailed motor examination that evaluates 14 items with 27 distinct functions. Each item is scored on a scale from 0 to 4. A total of 108 points are possible, with 108 representing maximal (or total) disability and 0 representing no disability.

In the finger taping test, subjects placed either hand palm down, fingers extended, with the index finger on a metal lever attached to a counting device. Individuals were told to press the level to actuate the counter and then encouraged to “warm up” at faster speeds until the correct technique was apparent to them. The warm-up was then conducted on the other hand. The Individuals were instructed to tap their index finger as quickly as possible for ten seconds, keeping the hand and arm stationary. This was followed by the alternate hand, and so forth until three trials were completed for each hand. The test takes approximately ten minutes to complete.

The PDQ-39 is a validated, often used Parkinson's disease measure of health status. It consists of thirty-nine questions, covering eight quality of life domains: Mobility, Activities of daily living, Emotional well-being, Stigma, Social support, Cognitions, Communication, and Bodily discomfort. A total score is also tabulated. It has been widely used by researchers all over the world.

### 2.2. Statistical Analysis

All analyses were conducted using SPSS 16.0. As a main analysis, four one way ANOVAs were conducted to determine if any between group differences existed between the exercise and control groups on any measure (UPDRS-III, BBS, Finger taping, or PDQ-39) at any time point (baseline, immediate intervention, or at four months). Statistical significance was set at the .05 level. As a secondary analysis, within group analyses were also conducted using repeated-measures *t*-tests on the same four dependent measures over the three time intervals.

## 3. Results

Participants were recruited via clinic flyers and word of mouth. Forty-one patients were initially consented, but due to an administrative error in applying exclusion criteria, five were dropped due to PD onset of less than three years and three were eliminated due to a MMSE of less than 23, leaving a total of 33 active participants. Of this group, three were dropped due to inability to get primary care physician clearance, one died, and six left for medical reasons (hip pain (2), knee pain (1), persistent fatigue (1), and missing exercise sessions (2)). Four participants (three in the control group and one experimental participant) failed to return for the four-month follow-up. Participants had a mean age of 68.2 years (SD = 8.8 years, range 46–88 years) and a mean time of PD onset of 7.2 years (SD = 6.2 years, range 3–29 years). Mean age and mean time of onset did not differ between the control and experimental groups. 

Mean scores for both groups on all measures at all three time intervals are included in Table 1. To analyze for a main effect, four one-way ANOVAs (SPSS 16.0) were calculated for the primary dependent measures (UPDRS-III BBS, Finger taping, PDQ-39) at the endpoint (four-month follow-up) to determine if significant treatment effects were present. Results for the UPDRS-III (*F* = 1.75, *P* = .20), BBS (*F* = .01, *P* = .98), finger tap right (*F* = .347, *P* = .56), finger tap left (*F* = .04, *P* = .84), and PDQ-39 (*F* = 2.02, *P* = .18) were all nonsignificant.

Similarly, the groups were examined using four one-way ANOVAs at the immediate postexercise point (8 weeks) to determine if differences existed. Results for the UPDRS-III (*F* = .06, *P* = .81), BBS (*F* = .001, *P* = .97) Finger tap right (*F* = .28, *P* = .60), Finger tap left (*F* = .26, *P* = .62) and PDQ-39 (*F* = 3.57, *P* = .07) were also all nonsignificant.

Within-subject effects were analyzed using ANOVA. Only the experimental group's UPDRS-III score demonstrated significant change over the course of the study (*P* = .04). Posthoc analysis, using paired *t*-tests, indicated the significance occurred between baseline and four-month posttesting, not between baseline and immediate posttesting. No other within-subject effects were found.

## 4. Discussion

No significant main effect differences were found between the control and exercise groups on any of four measures at the eight-week exercise point or at the four-month measurement interval. This is of importance as our investigation compared FE participants to a control group that had no specified exercise routine, creating a more marked contrast in exercise habits. Even with this heightened contrast no benefit on the measurement instruments was demonstrated. 

Our experimental group did show a significant within group improvement on UPDRS-III scores at the four-month period when compared to baseline. Improved UPDRS-III scores have been previously reported with FE participants [12, 13]. Interestingly, the current study failed to show improvement at the immediate postexercise period but did show improvement at the four-month follow-up point, whereas previous studies showed an initial improvement that dissipated over time [13]. The reason for our exercise participants showing a “delayed” improvement is counter-intuitive and requires further investigation.

This study roughly utilized sample sizes and similar aged participants to prior studies, but our participants had mean onset time of PD symptoms over seven years, whereas some [13] examined individuals with much shorter onset times. The longer duration of onset in our sample may have added to the general deconditioning effect seen with many individuals with PD. One possible hypothesis is that the positive results seen in other studies may be related to earlier exercise interventions. In addition, our sample had a large range of onset (26 years), creating the possibility that our sample was much more heterogeneous than other samples studied. We considered eliminating outliers but decided not to as the sample studied is very representative of the clinic patients we see daily.

As one of the first randomized, controlled investigations attempting to study the feasibility of utilizing an outpatient program of therapeutic exercise utilizing the Theracycle, this study revealed a number of issues that should be considered in future investigations. Although initial interest in the protocol was high, recruitment of subjects was an on-going challenge due to the duration of the study and the amount of commitment required. Economic factors also played a role as the cost to prospective subjects of twice weekly travel to the hospital was too much for many who were on limited incomes. This factor was greatly exacerbated by the upward spiral in national gasoline prices that occurred during the study period. 

Use of the Karnoven formula also proved problematic in those cases where subjects were taking beta-blockers, thus making it impossible to reach target heart rates. Use of an exertional formula, such as the Borg Rating of Perceived Exertion Scale or the “talk-test” [20], would likely be more utilitarian for this population. 

Due to both their age and the years of inactivity partially caused by PD, many prospective subjects were either exercise intolerant or incapable. The resultant overall patient recruitment pattern could have potentially led to an unknown selection bias with skewed results.

The PDQ-39 is the most widely used endpoint measure for persons with Parkinson's disease. However some [21] have noted limitations inherent to the instrument. Specifically attention has been drawn to the fact that the grouping of items into subscales is “overly complex” and its meaning “unclear.” With this in mind, we elected not to examine subscale scores, although a case could perhaps be made for doing so and we may have lost finer grain data. 

Finally, this study was limited by small sample size, as well as logistical issues and could be significantly improved upon with a larger sample size and better subject adherence to the study protocol, thus reducing selection biases in the current study.

## 5. Conclusions

Prior reports of FE as a benefit to persons with Parkinson's disease are intriguing, but this study failed to show significant treatment effects when compared to controls. Aside from a within subject improvement on UPDRS-III scores in the FE group no benefit was seen from forced exercise on the motor assisted cycle. Larger controlled studies are warranted, but our investigation highlights a number of clinical and logistical challenges that should be considered prior to undertaking such studies.

## Figures and Tables

**Table 1:** Mean scores (and SD) of control and exercise groups.

							Finger tap R					
	UPDRS-III score			BBS score			Finger tap L			PDQ-39 total score		
	Range = 0–108			Range = 0–56			Mean taps per 10			Range = 0–156		
	Time 1	Time 2	Time 3	Time 1	Time 2	Time 3	Time 1	Time 2	Time 3	Time 1	Time 2	Time 3
Experimental *n* = 13	15.7 (6.2)	14.2 (8.4)	10.4 (4.8)	47.6 (9.6)	46.8 (11.3)	48 (10. 4)	48.5 (8.8)	46.2 (11.4)	47 (10.4)	34 (22.3)	29.8 (23.6)	30.6 (20.8)
							44.2 (9.3)	38 (9.1)	40.5 (8.5)			
Control *n* = 10	16.9 (6.5)	15 (6.8)	14.1 (7.1)	44 (9.6)	46.7 (8.2)	47.9 (7.2)	42.8 (13.7)	43.2 (14.0)	43.4 (15.4)	62 (28.2)	50.3 (27.2)	48.7 (31.9)
							39.3 (14.4)	40.7 (14.7)	41.6 (14.5)			

Time 1: baseline; Time 2: immediate follow-up; Time 3: 4-month follow-up.

Higher UPDRS-III and PDQ-39 scores indicate more severe symptoms.

Time 3: three control participants and one experimental participant failed to return for follow-up testing.
